# Susceptibility-weighted imaging of cerebral fat embolism: A case report

**DOI:** 10.1097/MD.0000000000029462

**Published:** 2022-08-12

**Authors:** Xianwen Zhang, Liaoyuan Zheng, Jinfeng Duan, Shunyuan Zhang, Ying Zhou, Yufeng Tang

**Affiliations:** a Department of Neurology, Mianyang Central Hospital, School of Medicine, University of Electronic Science and Technology of China, Mianyang, China; b Department of Neurosurgery, Mianyang Central Hospital, School of Medicine, University of Electronic Science and Technology of China, Mianyang, China; c Department of Radiology, Mianyang Central Hospital, School of Medicine, University of Electronic Science and Technology of China, Mianyang, China.

**Keywords:** case report, cerebral fat embolism, coma, MRI, susceptibility-weighted imaging

## Abstract

**Rationale:**

Cerebral fat embolism (CFE) is a rare but critical disease in a clinical setting. Considering that manifestations and CT findings of CFE tend to be atypical, this condition is very difficult to diagnose. The purpose of this article was to assess the value of susceptibility-weighted imaging (SWI) in the diagnosis of CFE.

**Patient concerns:**

Our patient was an 80-year-old woman who developed hypoxemia, quadriplegia, and progressive confusion after fracture of the right femoral neck and right superior ramus of pubis within 24 hours.

**Diagnosis:**

T2-weighted magnetic resonance imaging (T2 W MRI), fluid-attenuated inversion recovery sequences, and diffusion-weighted imaging showed numerous hyperintense foci in the subcortex and white matter of both cerebral hemispheres, some of which were confluent and SWI showed multiple symmetrical punctate microhemorrhages in both hemispheres. Base on the history and MRI findings, the patient was diagnosed with CFE.

**Interventions:**

The patient received anticoagulation and lipid-lowering therapy.

**Outcomes:**

The patient regained consciousness, and her muscle strength in the limbs gradually recovered. One year after discharge, the patient could independently walk on her own.

**Lession:**

This case report shows the characteristics of CFE on SWI, which can help clinicians in diagnosing which can help clinicians in diagnosing CFE.

## 1. Introduction

Cerebral fat embolism (CFE) is an intracranial manifestation of fat embolism syndrome (FES), which is a critical disease rarely found in the clinical setting.^[1]^ As there are no characteristic signs in clinical presentation or routine brain imaging, CFE is difficult to diagnose.^[2]^ Following the development of MRI, it was found that susceptibility-weighted imaging (SWI) can reveal specific features of CFE, thus having the potential to be used as a new tool for diagnosing this disease.^[3]^ Herein, we reported on a case suffering from CFE and discussed the use of SWI in CFE.

## 2. Case presentation

**Figure 1.**
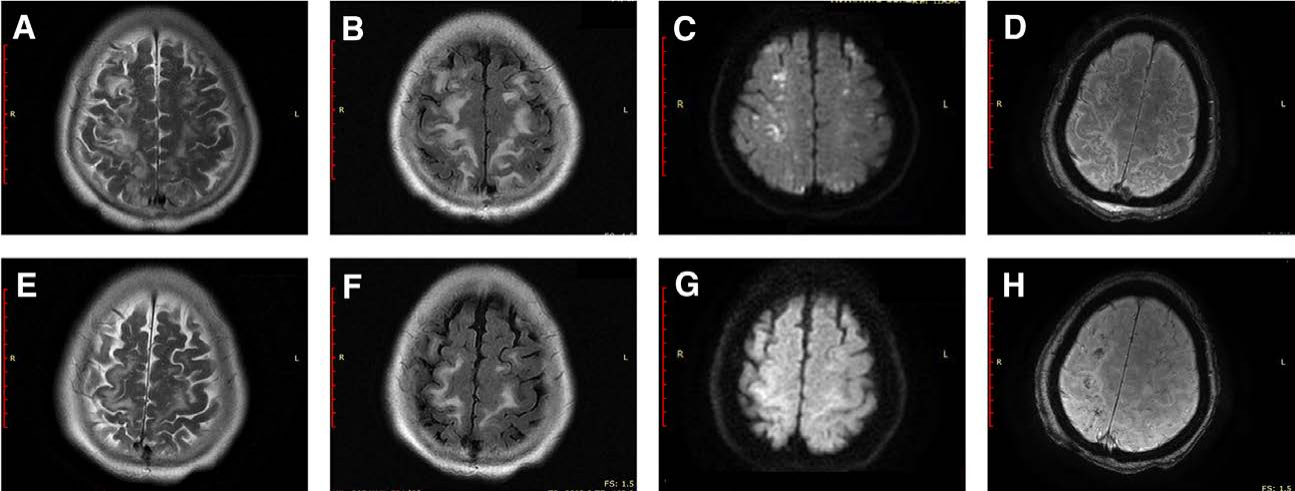
MR scans of the patient’s brain. T2W-MRI, FLAIR, DWI, and SWI sequences are shown from left to right: (A–D) are the images on the third day after onset, which showed extensive embolism lesions that spread along the bilateral cortices to the frontal, parietal cortex. Multiple pinpoint-like microhemorrhagic foci were seen in the lesion area, called “starry sky sign” on SWI. Images (E–H) were the reexamination images taken after 10 d. The scope of the lesions was smaller in (E–H) than 10 d before; microhemorrhagic lesions had a trend of fusion, and their overall number was reduced. DWI = diffusion-weighted imaging, FLAIR = fluid-attenuated inversion recovery, SWI = susceptibility-weighted imaging, T2 W MRI = T2-weighted magnetic resonance imaging.

A patient was an 80-year-old female who was sent to the Emergency Department on March 13, 2020, due to right hip pain with limited motion caused by a fall. Pelvic plain film radiography indicated right femoral neck fracture and right pubic ramus fracture. Ten hours after her fall, the patient gradually developed weakness in all limbs and poor mood without disturbances in speech, consciousness, sense, or defecation and without any fever or convulsion. Consequently, she was admitted to the Department of Neurology. At admission, her body temperature was 37.1°C, respiration was 20 times/minute, blood pressure was 170/100 mm Hg, heart rate was 86 beats/minute, and oxygen saturation was 86% to 88%. No skin petechiae were found, and chest breathing was predominant, with low breath sound in both lower lungs and fine rales in both lower lungs. Her cardiac results and abdominal physical examination were normal. External rotation of the right hip and swelling of 1/3 of the right thigh was noted. Neurological examinations revealed quadriplegia (right upper limb 1/5, right lower limb 0/5, left upper limb 2/5, and left lower limb 3/5) and bilateral positive Babinski sign. Glasgow Coma Scale (GCS) was 15 (E4V5M6) and National Institutes of Health Stroke Score was 12. The chest CT showed scattered exudation of the bilateral middle and lower lungs, and the brain CT was normal. Eight hours after admission, the patient’s consciousness progressively worsened, and she was in a shallow coma 24 hours after admission with GCS 9 (E2V3M4). Two days after admission, T2-weighted magnetic resonance imaging (T2 W MRI) (Fig. 1A) and fluid-attenuated inversion recovery (Fig. 1B) showed multiple high signal areas in the bilateral subcortical and white matter, some of which were fused into pieces. Diffusion-weighted imaging (Fig. 1C) showed partial high signal, and SWI (Fig. 1D) showed multiple microhemorrhages called “starry sky sign,” with obvious white matter involvement. Right echocardiography suggested the presence of patent foramen ovale and a small amount of shunting in the atrium. The patient was diagnosed with CFE. She received oxygen, 1500 ml of normal saline once a day, 5000 units of low molecular weight heparin calcium via subcutaneous injection every 12 hours and 20 mg of atorvastatin calcium once every night. The infusion speed was controlled at 2000 ml/day.

**Figure 2.**
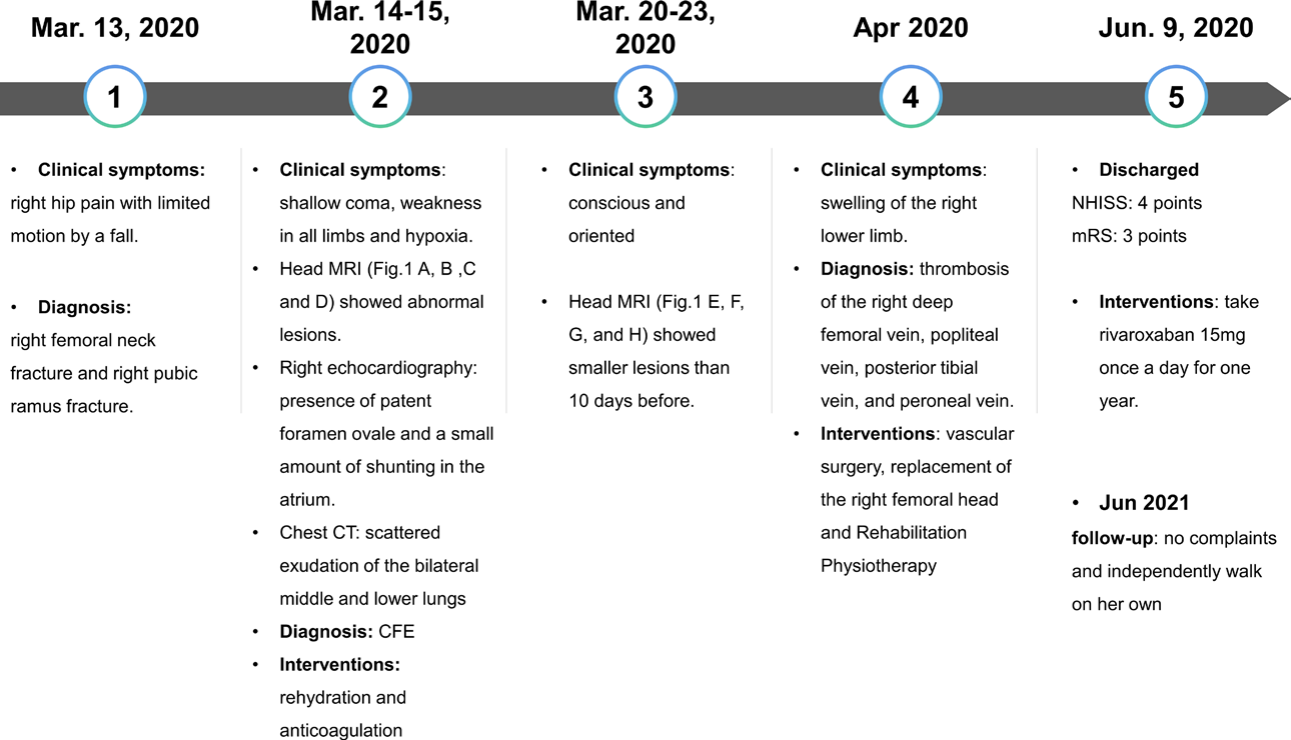
Timeline of relevant data of the diagnosis and interventions of the patient. mRS = Modified Rankin Scale, NIHSS = National Institutes of Health Stroke Score.

Seven days after admission, the patient was conscious and oriented and GCS was 15 (E4V5M6). Ten days after admission, the lesions on T2 W MRI (Fig. 1E), fluid-attenuated inversion recovery (Fig. 1F), diffusion-weighted imaging (Fig. 1G), and SWI (Fig. 1H) were smaller than 10 days before. Twelve days after admission, the patient presented with increased swelling of the right lower limb and a wider range of involvement. Color Doppler ultrasound of the lower limb vein revealed thrombosis of the right deep femoral vein, popliteal vein, posterior tibial vein, and peroneal vein. The patient was transferred to the vascular surgery department for percutaneous right femoral vein mesh implantation and venous thrombectomy. Thirty-seven days after admission, she was transferred to the orthopedic ward for replacement of the right femoral head. On June 9, 2020, the patient was discharged from the hospital. Her National Institutes of Health Stroke Score was 4, GCS was 15 and her Modified Rankin Scale was 3. She continued to take rivaroxaban 15 mg once a day for 1 year and was followed up every 3 months. In June 2021, the patient could independently walk on her own. The timeline of the patient, with relevant data on episodes and interventions, is presented in Figure 2.

## 3. Discussion

FES is a serious fracture-related complication, which usually occurs between 24 and 72 hours after injury, with a classic triad of respiratory manifestations (95%), cerebral effects (60%), and petechiae (33%).^[2]^ Neurological manifestations of FES can vary from mild cognitive changes to coma and even cerebral edema and brain death.^[4]^ In the early stage, CFE was considered to be the brain manifestation of FES; however, some patients showed mild systemic symptoms despite extensive intracranial embolization. Therefore, congenital patent foramen ovale, atrial septal defect, or pulmonary arteriovenous fistula are currently considered the anatomical basis of CFE. ^[5]^ Our case has patent foramen ovale as well.

The main diagnostic indicators of FES include hypoxemia (FiO_2_ > 0.4, PO_2_ < 60 mm Hg), central nervous system dysfunction, and ecchymosis. Secondary diagnostic indicators include tachycardia (heart rate > 120 beats/minute), fever (body temperature > 38.9°C), thrombocytopenia (platelets < 1.5 × 10^5^/L), fat globules in urine or sputum, retinal embolism, and unexplained anemia.^[6]^ Our patient had hypoxemia and a disturbance of consciousness. However, as the above clinical manifestations overlap with those of most trauma syndromes, it remains challenging to correctly diagnose FES. Due to the possibility of a right-to-left shunt in the heart and pulmonary arteriovenous fistula, some patients with CFE even lack the typical systemic manifestations of FES, so craniocerebral imaging is necessary to diagnose CFE. In the acute phase (1–4 days), the cranial MRI of CFE patients presents scattered lesions with limited diffusion on T2W, presenting a “starry sky sign.” The subacute phase (5–14 days) presents with cytotoxic cerebral edema in the periventricular and white matter fusion.^[7]^ SWI and gradient-echo imaging can reflect the dense multiple microhemorrhages (CMBs) in CFE lesions along with the white matter fiber bundles. In our case, multiple pinpoint-like microhemorrhagic foci were seen in the lesion area, spread along the bilateral cortices to the frontal-parietal cortex, called “starry sky sign” on SWI. The distribution of CMBs is characteristically different from diffuse axonal injury and cerebral amyloid angiopathy. CMBs of CFE are usually small and symmetrical, with diffuse distribution in white matter, cortex, basal ganglia, corpus callosum, and ventricles. CMBs in diffuse axonal injury are usually large and asymmetrical, located at the deep white matter and cutaneous medulla junction, corpus callosum, septum pellucida, hippocampus parietal lobe, and brainstem. CMBS in cerebral amyloid angiopathy are typically small and can be symmetric or asymmetric. They are mostly located in the lobule, cortex, and subcortex and do not affect the corpus callosum.^[4,8,9]^

Multiple treatment options for FES have been evaluated in the past without significantly changing clinical outcomes, including clofibrate, dextran-40, ethyl alcohol, heparin, aspirin, human albumin, and steroids.^[2]^ Although anticoagulant therapy is not required for CFE, restricted passive limb movement caused by fractures, bed rest caused by consciousness disorders, and hypoxemia are high-risk factors for deep vein thrombosis. In this case, the patient developed extensive deep vein thrombosis, for which she received anticoagulant therapy. The overall mortality of FES was 5% to 15%. Dyspnea is the common cause of death. Secondary pulmonary infections and deep vein thrombosis are common complications.^[1,10]^ In the present case, hypoxemia lasted for several days.

## 4. Conclusion

In conclusion, manifestations of CFE are diverse, and the clinical outcome is rapidly changing. SWI can quickly identify the characteristic “starry sky sign” microhemorrhagic lesions of CFE; thus, its diagnostic imaging value in CFE is greater than that of conventional MRI or CT, which makes it worthy of clinical application.

## Author contributions

All authors have read and approved the final manuscript.

Data curation: Shunyuan Zhang, Ying Zhou.

Project administration: Yufeng Tang.

Writing – original draft: Jinfeng Duan, Xianwen Zhang.

Writing – review & editing: Liaoyuan Zheng, Xianwen Zhang.
